# Agar Gel as a Non-Invasive Coupling Medium for Reflectance Photoacoustic (PA) Imaging: Experimental Results on Wall-Painting Mock-Ups

**DOI:** 10.3390/jimaging8090235

**Published:** 2022-08-30

**Authors:** Antonina Chaban, George J. Tserevelakis, Evgenia Klironomou, Giannis Zacharakis, Jana Striova

**Affiliations:** 1 National Institute of Optics—Italian National Research Council, Largo E. Fermi 6, 50125 Florence, Italy; 2 Institute of Electronic Structure and Laser, Foundation for Research and Technology Hellas, Plastira 100, Vassilika Vouton, 70013 Heraklion, Crete, Greece

**Keywords:** agar gel, wall painting, photoacoustic, non-invasive

## Abstract

The new reflectance set-up configuration extended the applicability of the photoacoustic (PA) imaging technique to art objects of any thickness and form. Until now, ultrasound gel or distilled water have been necessary as coupling mediums between the immersion-type transducer and the object’s surface. These media can compromise the integrity of real artwork; therefore, known applications of reflectance PA imaging have been limited to only experimental mock-ups. In this paper, we evaluate an alternative non-invasive PA coupling medium, agar gel, applied in two layers of different consistency: first, rigid—for the protection of the object’s surface, and second, fluid—for the transducer’s immersion and movement. Agar gel is widely used in various conservation treatments on cultural heritage objects, and it has been proven to be safely applicable on delicate surfaces. Here, we quantify and compare the contrast and signal-to-noise ratio (SNR) of PA images, obtained in water and in agar gel on the same areas, at equal experimental conditions. The results demonstrate that the technique’s performance in agar is comparable to that in water. The study uncovers the advanced potential of the PA approach for revealing hidden features, and is safely applicable for future real-case studies.

## 1. Introduction

Non-invasive and non-contact revealing of hidden features in multi-layered wall paintings is of crucial importance in heritage science and art history. Recently, a set of analytical tools available for this purpose has been enriched by a novel reflectance prototype of the photoacoustic (PA) imaging technique [1,2]. Scientific studies on experimental mock-ups have shown that a combined application of photoacoustic and multispectral imaging techniques could reveal various hidden underdrawings in the multi-layered systems of up to 200 μm thickness [2]. Specifically, charcoal and graphite outlines were detected under gypsum, limewash, and pigmented layers, with the latter applied as a fresco or a secco (egg yolk binder). It was also observed that the image contrast related to covered charcoal and graphite revealed by PA imaging was higher than in the images obtained by a multispectral camera, with spectral sensitivity up to 1100 nm [1].

However, the reflectance PA set-up in its available prototype configuration is characterized by a series of limitations that are currently being worked on, constantly improved, and solved. This paper illustrates progress in the applicability of the reflectance photoacoustic imaging technique in a completely non-invasive way. Until now, ultrasound gel or distilled water have been commonly exploited as coupling mediums between the transducer and the object’s surface [1,2,3,4,5,6,7]. Although beneficial for the ultrasound wave propagation, such transducer immersion media can become harmful to the delicate pictorial surfaces, especially over a prolonged time. For this reason, until now, the applicability of this novel technique was limited to experimental wall painting mock-ups.

Here, we introduce and evaluate the applicability of agar gel as both a surface protectant and a coupling medium for the reflectance photoacoustic imaging technique. Agar gels are widely used in the conservation of cultural heritage (CH) objects, due to their versatile effectiveness in cleaning and non-invasiveness towards the delicate surfaces (easel and wall paintings, stone, wood, paper, gypsum substrates etc.) [8,9,10,11,12,13,14,15,16]. The strong water retention properties minimize the penetration of water into the substrate. Agar is a natural polysaccharide extracted from several species of red seaweeds, able to form semi-rigid, thermo-reversible, and hydrophilic gels [16]. When boiled with water in a concentration of 0.5–5%, it produces a colloidal solution that jellifies on cooling at around 35 °C [17]. It can be used in different formulations: jellified and cold—suitable for positioning on a flat surface; still warm (35–40 °C) and fluid—suitable for pouring on a surface relief; or otherwise milled until it reaches a snow-like consistency—suitable for pressing as a pad onto the surface of any shape [8,9,10,11,12,13,14,15,16,17,18]. We examine and compare different formulations and step-by-step procedures for agar gel formation, according to their suitability as surface protectants and their specific effectiveness as PA coupling media. The latter is carried out by comparing the performance of the reflectance PA technique in agar and in distilled water. In specific, we examined the image contrasts and signal-to-noise ratio in the PA scans.

## 2. Materials and Methods

### 2.1. Materials

The examined wall-painting mock-up represents some common paint layer stratigraphy occurring in historical wall painting, including the following: secco painting using an egg yolk binder (1), gypsum (2) and limewash cover (4), as well as fresco painting (6,7,8), and a combination of fresco and secco painting techniques (9). The examined cases range from simple (one single coat) to a maximum of four nonhomogeneous layers, with/without a final varnish coat. One layer corresponds to one passage by a brush. The detailed description of the mock-ups discussed in this paper after examination by reflectance PA prototype in distilled water and in agar can be found in Table 1.

#### 2.1.1. Wall Painting Support

A lightweight wood fiber panel (Celenit) served as a support. The application of mortar and fresco paint layers were performed following the rules of the traditional fresco painting technique [19,20,21,22,23] at the Accademia dell’Affresco in Padua (Italy). The fresco mortar (max 15 mm thick) was applied in two subsequent layers: (1) *arriccio*, a thick layer (10–12 mm) of medium coarse mortar (a mix of slaked lime and medium grain sand (1:2) with water); (2) *intonachino*, a thin layer (2–3 mm) of fine coarse mortar (a mix of slaked lime and fine grain sand (1:2) with water).

#### 2.1.2. Underdrawings

The underdrawings with sinopia and charcoal pigments (purchased from Dolci, Verona) were applied on the wet *intonachino* surface using the traditional *spolvero* (pouncing) technique, characteristic of fresco painting [19]. The dots were immediately outlined by a brush soaked with pigment in water. The graphite drawings were performed by pencil (Koh-i-noor Hardmuth, 2B) directly on the dried *intonachino*, following the secco wall painting technique.

Sinopia, or red earth, is predominantly composed of hematite, a dehydrated form of iron oxide [24]. Charcoal, or simply carbon black (commercial name: *nero carbone*), is the product of charring wood, which contains amorphous carbon and other impurities. Graphite contains a crystalline form of carbon [25,26].

#### 2.1.3. Hiding Layers

Hiding layers include several pigmented (fresco and secco, using tempera) and covering layers (limewash, gypsum). The pigments used for fresco painting include yellow ochre (iron oxide hydroxide, α-FeOOH) and Egyptian blue (copper calcium silicate, CaCuSi_4_O_10_). All the pigments were purchased from Dolci and Zecchi (respectively, Verona and Florence, Italy). Tempera overpaints for the secco were applied by brush using Egyptian blue or yellow ochre, mixed with egg yolk binder (egg yolk and distilled water, in proportion 1:1), with a 40–70 µm thickness, as detailed in [2]. Covering layers of limewash and gypsum, 60–80 µm thickness, as described in [2], were applied by brush, using rabbit glue for gypsum (CaSO_4_.2H_2_O, glue/water proportion 1 g:12 mL), and adding a small amount of low-fat milk to the limewash.

#### 2.1.4. Varnish

The varnish was prepared by dissolving shellac flakes in ethanol (25 wt%) for 48 h, and then it was applied by brush in two passages, with intermediate complete drying. Shellac resin flakes were purchased from Phase Srl (Florence, Italy).

The PA results in water for samples 1, 2, and 4 were presented and discussed in our previous work [2]. The PA results in water and in agar for samples 6, 7, 8, and 9, and the results in agar for samples 1, 2, and 4, are presented and discussed here for the first time.

### 2.2. Methods

#### 2.2.1. Reflectance Photoacoustic Imaging

The detailed technical description of the epi-illumination PA imaging prototype that was utilized in the context of this study can be found elsewhere [1,2]. A pulsed nanosecond NIR beam at 1064 nm is employed for the efficient excitation of PA signals from the hidden dark pigments, following its loose focusing on the surface of the mock-up. The laser-induced ultrasonic waves are subsequently transmitted through the hiding layers and the coupling medium prior to their detection by a single-element spherically focused piezoelectric transducer. The signals are amplified, digitized, and recorded by a fast oscilloscope during the mechanical raster-scanning of the sample by a XY motorized stage, to attain a point-by-point data acquisition which is synchronized with the trigger signal of the laser source. The PA waveforms are averaged two times for signal-to-noise ratio (SNR) improvement, transferred to a computer, and bandpassed between 100 kHz and 30 MHz before the estimation of the peak-to-peak amplitude value, providing the contrast of the images. The applied fluency in the described experiments ranged from 0.44 to 1 mJ/s, whereas the pixel size is equal to 300 × 300 μm^2^. The total time required for the recording of the PA scans ranged from 1 to 3 h (for scanned areas with horizontal dimensions ranging from 15 × 15 to 20 × 25 mm, respectively).

#### 2.2.2. Coupling Media for Ultrasound Wave Propagation

Each sample was placed at the bottom of a 3D-printed sample holder. As immersion media for the efficient propagation and subsequent detection of PA signals, we used distilled water and agar gel, evaluated in this study. Agar gel, in powder form, was purchased from Sigma Aldrich (now Merck KGaA). In an attempt to both protect the depicted surface and to allow the complete and constant PA transducer immersion and movement, different concentrations (0.25, 0.3, 0.5, 1, and 2 wt% agar solution in water) and preparation procedures were evaluated (variable number (1–4) of agar heating/cooling cycles) and preparation conditions (variable air temperature, stirring, etc.).

## 3. Results

### 3.1. Initial Validation of Reflectance PA Imaging in Agar

As the first step, we carried out the preliminary PA tests through agar gel on uncovered graphite pencil drawings. These tests were aimed to verify the general applicability of agar as an ultrasound coupling medium and to select the optimal agar gel formulation. We observed that none of the single formulations of the agar gel was capable of meeting both operational and protective requirements. To enable the ultrasound detection, (a) the agar layer should remain fluid and flexible, because the transducer needs to be fully immersed in the coupling medium during the scanning movements; (b) the agar layer in contact with the wall surface should be solid to guarantee a good degree of water retention. We found a solution to this problem by applying the agar in two subsequent layers, varying the agar powder concentration in water, as well as its cooling conditions.

The first layer, prepared by dissolving agar in distilled water at 2 wt%, was heated up to 90 °C three times and cooled down under laboratory conditions (i.e., 25 °C). During the last cooling cycle, at a temperature of around 35 °C, the fluid agar gel was applied directly onto the wall painting surface to avoid formation of air bubbles during jellification.

**Figure 1:**
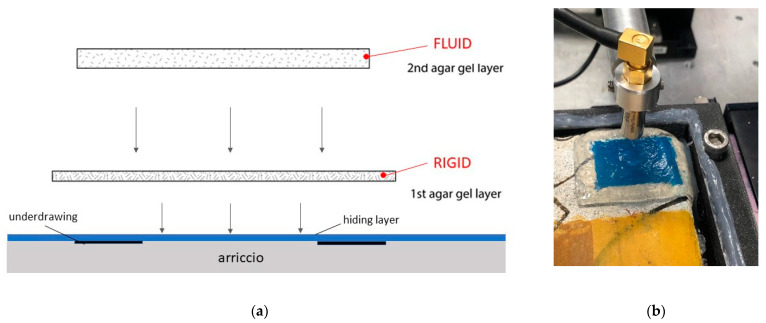
Scheme of agar gel double-layer application (**a**) and visible image illustrating the PA reflectance imaging operation in agar (**b**).

The second layer, prepared by dissolving agar in distilled water in our experiments at 0.25, 0.3, and 0.5 wt%, was heated up to 90 °C two times and cooled down under laboratory temperatures, while stirring continuously. Upon reaching the laboratory temperature (25 °C), the agar gel, which maintained a fluid consistency due to stirring, was applied over the first (solid and protective) agar gel layer. All the three agar gel concentrations (0.25, 0.3, 0.5 wt%) enabled a good transmission of the PA signal to reveal the uncovered graphite drawing on the lime mortar. We selected the formulation with 0.3 wt% agar concentration, which assured a constant immersion of the transducer during scanning movements and a stable in-place hold of the gel on the surface. The scheme of the developed agar application system and a photo illustrating the applied agar gel for reflectance photoacoustic imaging operation are shown in Figure 1a,b, respectively.

### 3.2. Reflectance PA Imaging in Agar vs. Water

Having verified the feasibility of agar gel as a coupling medium for an efficient PA imaging, we proceeded with transposing the methodology to image the underdrawings covered by different hidings (limewash, gypsum) and single/superimposed pictorial layers: fresco and secco (egg yolk tempera). All the results in agar were compared to the results in water.

**Figure 2:**
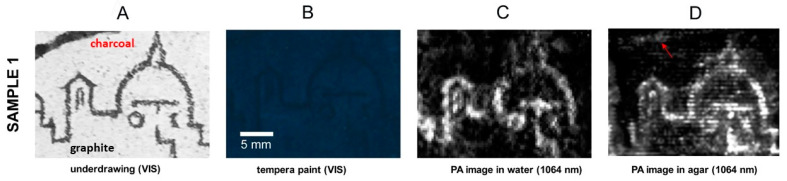
Reflectance PA imaging results on graphite and charcoal underdrawings (**A**) hidden under Egyptian blue tempera paint (**B**); comparison of results in water (**C**) and agar (**D**). The red arrow indicates the area corresponding to the charcoal outline in a PA image. The scale in 2B is valid for all images in the row.

In sample 1, two types of carbon-based pigments were hidden under a thick layer of Egyptian blue egg yolk paint, as shown in Figure 2A,B. As reported in the previous work performed on this sample in water [2], only the graphite line was revealed in those measurements (Figure 2C). It was explained by a stronger absorption at 1064 nm of crystalline carbon in graphite in comparison to amorphous carbon with impurities in charcoal [25,26,27]. During the second step of investigation, namely in agar gel, we detected an additional low signal of charcoal, along with the expected strong response from the graphite drawing (Figure 2D). The PA images were processed using the same methods, applying the function of 1.0% contrast stretching in ImageJ.

**Figure 3:**
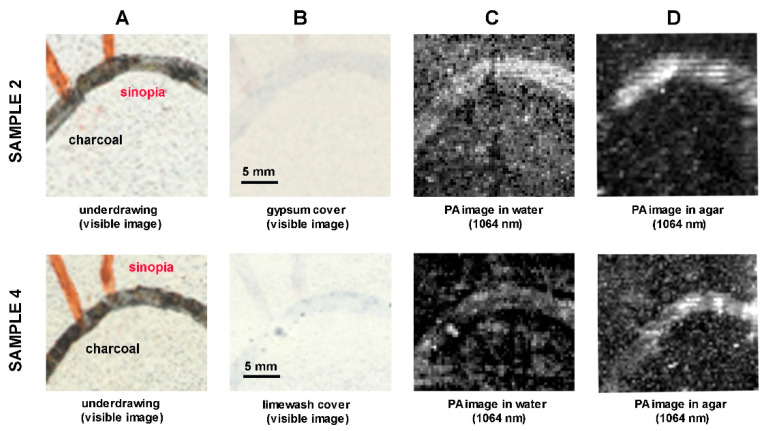
Reflectance PA imaging results on graphite and charcoal underdrawings (**A**) hidden under Egyptian blue tempera paint (**B**); comparison of results in water (**C**) and agar (**D**). The red arrow indicates the area corresponding to the charcoal outline in a PA image. The scale in 3B is valid for all images in the row.

The PA results on gypsum/limewash covered underdrawings are shown in Figure 3. Column A shows the visible image of the underdrawings, and column B shows the visible image of underdrawings after being covered; columns C and D illustrate the PA images, acquired in water and agar, respectively. In all the results, shown in column C and D, we observe that the charcoal lines, optically absorbing at 1064 nm, are revealed in both coupling media. Sinopia lines are not revealed in either water or agar, as expected due to the low optical absorption of this material at 1064 nm [24].

The PA results on gypsum/limewash covered underdrawings are shown in Figure 3. Column A shows the visible image of the underdrawings, and column B shows the visible image of the underdrawings after being covered; columns C and D illustrate the PA images, acquired in water and agar, respectively. The PA images are shown after processing in ImageJ: by applying the function of 1.0% contrast stretching and 0.6 gamma correction to the PA images of sample 2, and the function of 1.0% contrast stretching and 0.9 gamma correction to the PA images of sample 4. In all the results, shown in column C and D, we observe that the charcoal lines, optically absorbing at 1064 nm, are revealed in both coupling media. Sinopia lines are not revealed in either water or agar, as expected due to the low optical absorption of this material at 1064 nm [24].

**Figure 4:**
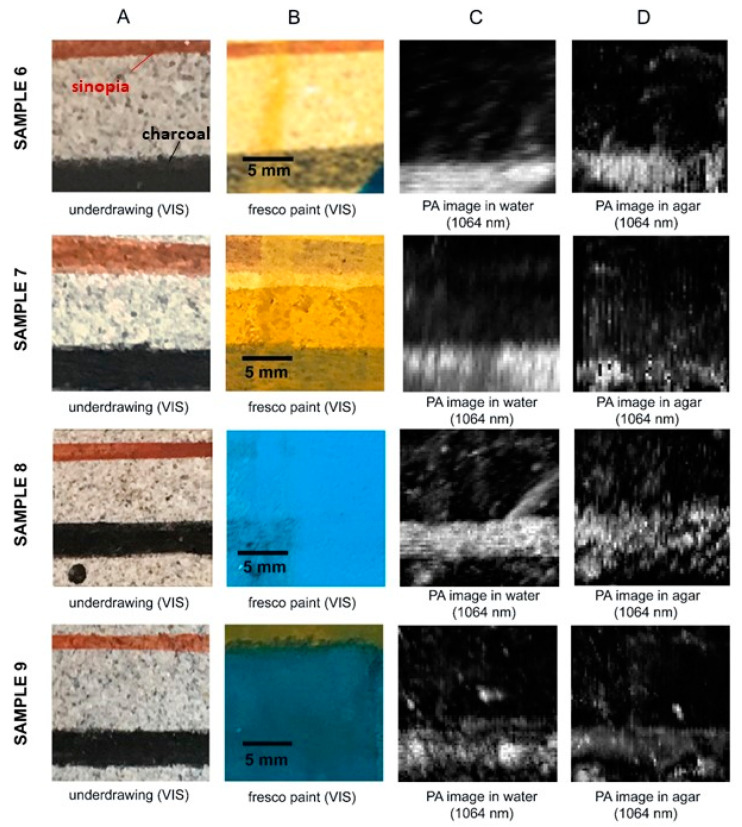
Reflectance PA imaging results on charcoal and sinopia underdrawings (**A**), hidden under pigmented fresco and egg yolk tempera layers. Comparison of experimental results in distilled water (**C**) and in agar gel (**D**). The scale in (**B**) is valid for all images in the row.

The detectability of the underdrawings in the PA images of fresco and secco painted samples 6–9, scanned at equal conditions and postprocessed using the same method (contrast stretching 1.0% in ImageJ), is more clear in water than in agar, as evident in Figure 4. This could be related to the incomplete adherence of the first agar gel layer to the varnished (6,7,9) or unvarnished (8) rough pigmented surface and the consequent presence of small air gaps. The latter can compomise the detectability of the PA signal. Nonetheless, the strongly absorbing features, i.e., charcoal lines, are revealed both in water and in agar, in the presence of 1 up to 4 hiding paint layers. Furthermore, the detectability of all the charcoal lines, either in water nor in agar, is compromised by the upper coat of shellac varnish.

### 3.3. Image Contrast (CI) Ad Signal-to-Noise Ratio (SNR) Evaluation of Reflectance PA Imaging in Agar and in Water

In order to quantitatively evaluate the quality of the results obtained in two coupling media, i.e., distilled water and agar gel, image contrast (CI) values and signal-to-noise (SNR) ratios were compared for PA scans, obtained using equal experimental conditions (samples 6–9). For this purpose, we initially contrast stretched all the PA images by saturating 0.3% of the pixels. First, we calculated the respective Michelson contrast values [28,29], on five selected representative pixel brightness profiles perpendicularly to the sketch lines. For each measured profile, the contrast value C was calculated using Equation (1):

***C*** = ***Aline*** − ***Abackg***/***Aline*** + ***Abackg*** (1)

where Aline and Abackg correspond to the average brightness value of the pixels representing, respectively, the underdrawing line and the fresco mortar background. The final contrast for each image was calculated as the average of the five selected profiles, to compensate for potential local signal variabilities.

For the calculation of signal-to-noise ratio (SNR) [30,31], we selected three equal rectangular areas (200 × 200 pixels each) on the signal areas and on the background of the PA images. For each compared signal/background set, the SNR was calculated using Equation (2):

*SNR* = *μ*/*σ*, (2)

where *μ* is the mean value of the signal and *σ* is the standard deviation of the background signal.

The standard error of the mean (SEM) was calculated using Equation (3):

*SEM* = *σ*/*√n*, (3)

where *σ* is the standard deviation of the calculated value and √*n* is the square root of the number of calculated values.

**Figure 5:**
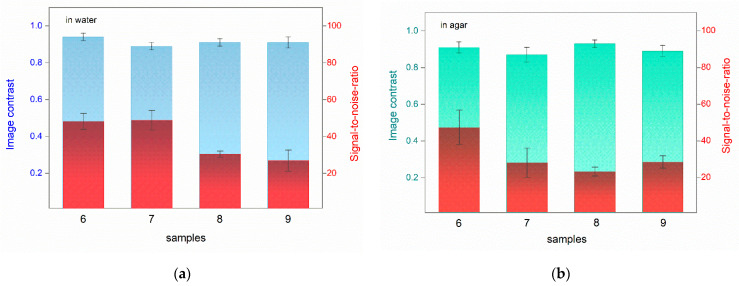
Comparison of CI and SNR values in PA images of all samples acquired in: (**a**) water; (**b**) agar. The left axis reports CI values (blue for water and green for agar), and the right axis reports SNR values (red for both water and agar).

The CI and SNR results in water and in agar are plotted in Figure 5a,b, respectively.

We can observe that the image contrast values of the PA images, acquired in agar gel, are comparable to those obtained in distilled water. Specifically, in water, the CI values range from 0.89 (±0.04) to 0.94 (±0.05), while in agar gel, the values range from 0.87 (±0.04) to 0.93 (±0.03). The SNR values are in the range of 27 to 59 for the measurements in water and in the range of 23 to 47 for the measurements in agar. Slightly higher standard error values in agar are observed for samples 6 and 7. The differences can be related to one or more factors, e.g., the incomplete adhesion of the agar gel to the hydrophobic shellac varnish and/or the intrinsic roughness of the pigmented surface, or due to the quality of the agar gel. Both in distilled water and in agar gel, the CI and SNR values are not compromised by the presence of a hydrophobic shellac coat (samples 6, 7, 9), nor by an increased number or variety of hiding layers (sample 9).

## 4. Discussion

The substitution of distilled water with the agar gel coupling medium shows promising results, representing an important step towards the applicability of the PA technique in the real wall-painting cases. Being comparable in terms of CI and SNR values, the specific performance of reflectance PA imaging using agar gel may depend on a series of factors. Among those influencing the adhesion of gel to the surface, we can list the fresco mortar surface topography and roughness (including the grain size of the pigment, sand, and calcium carbonate, etc.). An important issue for good signal propagation is also the quality of the prepared agar gel, which should be free of air bubbles and as homogeneous as possible. The latter improves with the number of heating/cooling cycles (the optimum number of cycles is 3). another factor to be considered is the conservation state of the hidden underdrawings and of the substrate itself. In this respect, to safeguard the pictorial surface, it might be beneficial to perform preventive consolidation prior to the PA measurements using agar. Indeed, the performed measurements show that the presence of a thin organic layer, e.g., shellac, does not significantly influence the quality of the obtained results in agar.

## 5. Conclusions

We developed a double-layer agar gel system for operating a novel reflectance PA imaging prototype on precious wall-painting surfaces. We assessed the performance of agar gel in terms of signal propagation and detection properties, as well as in terms of non-invasiveness towards the delicate surfaces of fresco and secco wall paintings. When comparing to previously used media, i.e., distilled water, the use of agar proved to provide a comparable image contrast and signal-to-noise ratio, at the same time acting as a surface protectant during the non-invasive examination of underdrawings and hidden features. More work is in progress. However, as far as it can be concluded from these preliminary experimental results, a double layer of agar gel should be applicable on varnished and—if solidified—even on well preserved, completely dried (at least 2 years drying at natural conditions), flat unvarnished pictorial surfaces. The proposed methodological advancement, based on the use of agar gel as a new PA coupling medium, significantly broadens the applicability of the technique in the heritage science field. Our further steps include the development of a measurement configuration for use on vertical surfaces and finally, the application of the reflectance PA technique on wall-painting real-case studies.

## Figures and Tables

**Table 1:** Mock-up paint layer stratigraphy.

Sample Type		Sample Number	Underdrawing	Hiding Layer	Layers	Varnish
**Covered**		2	sinopia	gypsum	1	x
			charcoal	gypsum	1	x
		4	sinopia	limewash	1	x
			charcoal	limewash	1	x
**Painted**	fresco	6	sinopia	yellow ochre	1	shellac
			charcoal	yellow ochre	1	shellac
		7	sinopia	yellow ochre	2	shellac
			charcoal	yellow ochre	2	shellac
		8	sinopia	egyptian blue	3	x
			charcoal	egyptian blue	3	x
	tempera	1	graphite	egyptian blue	3	x
			charcoal	egyptian blue	3	x
	fresco + tempera	9	sinopia	egyptian blue + yellow ochre	2 + 2	shellac
			charcoal	egyptian blue + yellow ochre	2 + 2	shellac
